# Developmental Structural Tooth Defects in Dogs – Experience From Veterinary Dental Referral Practice and Review of the Literature

**DOI:** 10.3389/fvets.2016.00009

**Published:** 2016-02-08

**Authors:** Sonja Boy, David Crossley, Gerhard Steenkamp

**Affiliations:** ^1^Department of Oral Pathology, Faculty of Health Sciences, Sefako Makgatho Health Sciences University, Pretoria, South Africa; ^2^Division of Oral Surgery, Animal Medical Centre Referral Services, Manchester, UK; ^3^Department of Companion Animal Clinical Studies, Faculty of Veterinary Science, University of Pretoria, Pretoria, South Africa

**Keywords:** tooth abnormalities, enamel hypoplasia, gemination, fusion, concrescence, odontogenesis

## Abstract

Developmental tooth abnormalities in dogs are uncommon in general veterinary practice but understanding thereof is important for optimal management in order to maintain masticatory function through preservation of the dentition. The purpose of this review is to discuss clinical abnormalities of the enamel and general anatomy of dog teeth encountered in veterinary dental referral practice and described in the literature. More than 900 referral cases are seen annually between the two referral practices. The basis of the pathogenesis, resultant clinical appearance, and the principles of management for each anomaly will be described. Future research should be aimed toward a more detailed analysis of these conditions so rarely described in the literature.

## Introduction

Developmental abnormalities of teeth, albeit uncommon in general veterinary practice, are seen with higher frequency in the dental referral clinic. Understanding these abnormalities is important for optimal management in order to maintain masticatory function through preservation of the dentition, while improvement of the clinical appearance should be of secondary importance. Many detailed studies on all types of dental abnormalities affecting humans are available in the literature, but reviews of dental anomalies in dogs are scant (1–3), and little cognizance is given to the etiopathogenesis of these defects. Developmental pathology of teeth may be classified in several ways, but categorization according to abnormalities in the number, size, and shape or clinical structure of teeth is a clinically orientated approach frequently utilized by veterinary dentists. For the purpose of this review, abnormalities of enamel structure and macroscopic tooth anatomy encountered in two veterinary dental referral practices, one in South Africa and the other in the United Kingdom will be discussed. More than 900 dental referral cases were seen annually between the two practices over a 20-year period, and cases were carefully documented during that time. The basis of the etiopathogenesis and resultant clinical appearance as well as the management principles of each anomaly will be described.

The structural morphology and anatomic features of teeth are established during the initiation and morphogenetic or bell stage of tooth development (4). All variations or abnormalities in any of these features can therefore be explained through a comprehensive understanding of the process of tooth development formally known as odontogenesis.

## Odontogenesis

**Figure 1:**
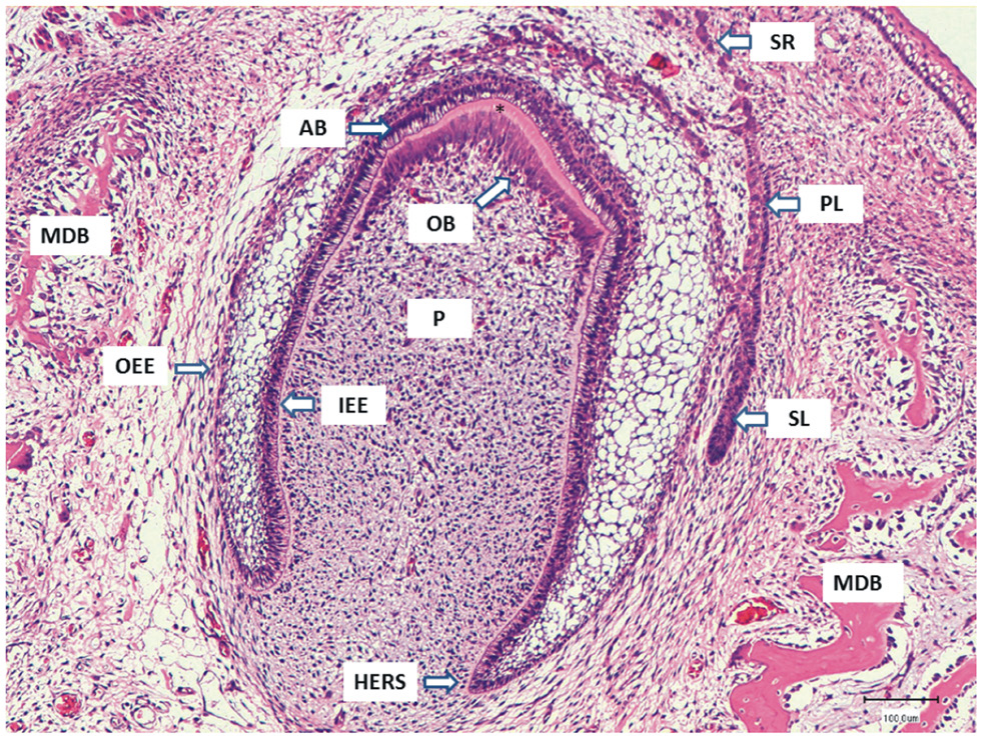
**The micrograph represents a deciduous tooth germ in the developing mandible (MDB) of a dog fetus**. The primary dental lamina (PL) is breaking up to form the small epithelial rests of Serres (SR), and the successional dental lamina (SL) for the permanent tooth is developing next to the deciduous tooth germ. The inner enamel epithelium (IEE) gives origin to the enamel-producing ameloblasts (AB) seen as tall cylindrical cells with prominent subnuclear vacuolization. The odontoblasts (OB) that developed from the papilla mesenchyme have already formed a layer of dentin (asterisk) and are moving toward the developing pulp (P). The IEE and outer enamel epithelium (OEE) come together at the Herwig root sheath (HERS) where the tooth root formation will take place.

During embryological development, a group of cells, the neural crest cells, separate from the developing neural tube, migrate, differentiate, and go through epithelial to mesenchymal transformation. This process is responsible for the development of the enriched connective tissue of the head and neck also known as ectomesenchyme (5–7). With the exception of the enamel and some cementum, the tissue of the tooth originates from the ectomesenchyme (5–7). Odontogenesis is initiated with the formation of a band of thickened oral epithelium, which gives origin to the dental laminae, thin down growths of epithelium extending from the overlying epithelium (Figure 1) into the developing jaw bone (6, 8). Sequential and reciprocal interactions between these epithelial strings and its surrounding condensed ectomesenchyme results in tooth development, seen as a series of morphological phases named bud, cap, and bell stages (7–10). The cap stage of odontogenesis is represented by epithelial cells in the form of a cap resting on a bed of condensed ectomesenchyme. The latter will eventually form the dental papilla and the meshwork of ectomesenchyme, which surrounds the cap, and its developing papilla will give origin to the dental follicle (6, 7). The odontogenic epithelium and the ectomesenchyme surrounding it are collectively termed the tooth germ or dental organ (6–8). The epithelium of the tooth germ continues to proliferate until it resembles a bell, now known as the bell stage of odontogenesis. This marks the phase of histo- and morphodifferentiation of developing teeth during which time the form of the crowns as well as the number, size, and shape are determined (4). The epithelial component of the enamel organ at that time consists of outer enamel epithelium (OEE), inner enamel epithelium (IEE), and stellate reticulum (SR) (7, 11). The OEE and IEE are now separated by SR over the forming crown becoming confluent at the cervical loop. Root formation will proceed through apical proliferation of these two layers at the cervical loop, this area being known as the Hertwig’s epithelial root sheath (HERS) (6, 8). Differentiation of the IEE will give origin to the enamel-producing ameloblasts (AB), against which the peripheral cell layer of the dental papilla ectomesenchyme will form dentin-producing odontoblasts (12). Differentiation and hard tissue formation starts at the tip of the developing cusp and proceeds apically, creating the form of the crown. The tooth forming dental lamina eventually breaks up into distinct epithelial islands known as the rests of Serres (7) (Figure 1). Although thousands of genes play a role in the process of odontogenesis (13), no tooth-specific regulatory genes have as yet been discovered (14).

Any interference with the process of odontogenesis will result in structural abnormalities of the enamel and/or the gross anatomy (or form) of a developing tooth. Disturbances earlier in the process of tooth development will result in more extensive involvement and abnormality in the clinical appearance of the tooth than disturbance later in this process when certain aspects of the tooth will have already developed normally. Consideration of the times of development, mineralization, and eruption of each tooth is therefore necessary in order to understand and explain the clinical pathology. In humans, several years elapse between the initial mineralization of the developing tooth crown and the time when the crown is completed and ready for eruption (7). There is also a very wide time difference between the development and eruption of different teeth within the dental arches (7). Structural anomalies affecting an area of a specific tooth or teeth can therefore be relayed back to a specific age-related incident almost to the week of development. However, in the case of dogs, hard-tissue formation of all permanent teeth occurs within a narrow timeframe of approximately 8 weeks starting shortly after birth (15). An accurate individual tooth mineralization schedule for dogs is needed in the veterinary dental literature. Jaw growth and development of alveolar bone occur around the forming teeth. These have then to move through the bone, emerging through the gingival, and erupting into a functional position if all proceeds normally. The timing of tooth emergence varies between dog breeds, typically being quoted as ranging from 12 to 16 weeks for incisors, 16 to 24 weeks for canines and premolars, and 20 to 32 weeks for molars (2). No large-scale studies have been performed to provide accurate figures for particular breeds. It is well established that systemic diseases in dogs, particularly those associated with severe pyrexia, infection with epitheliotropic viruses, exposure to certain drugs (such as tetracycline or high levels of systemic fluoride) during tooth development, can result in enamel abnormalities (16). It has been the experience of the authors that, in dogs, such occurrences happening up to 8 weeks of age result in abnormalities of several or all teeth, the extent of which is dependent on the stage of tooth development at the time and duration of the insult. In addition to systemic diseases, local inflammation, trauma to a developing tooth germ, and a number of genetic disorders can all result in clinically abnormal enamel and/or tooth morphology, either occurring sporadically or as part of developmental syndromes. Local infection or trauma to developing canine and carnassial teeth as late as 12 weeks of age may still result in enamel defects.

### Abnormalities of Enamel Structure

The development of enamel, or amelogenesis, is a highly regulated and complex developmental process involving numerous genes and their products (17). ABs are responsible for the formation of a unique extracellular matrix, matrix maturation, and its mineralization to form enamel. An optimal extracellular environment is required for this to proceed normally (7, 18). ABs within the developing tooth germ are very sensitive to external stimuli, and many factors may disturb matrix formation and secretion by these specialized cells, or have a negative influence on enamel matrix mineralization. Unlike bone, dentin, or cementum, there is no process of remodeling or repair after enamel formation has been completed, and disruption in its formation leaves a permanent lesion in the ensuing tissue (19). In the referral practices of the authors, developmental enamel abnormalities in dogs mostly occur as various forms of enamel hypoplasia (EH).

#### Enamel Hypoplasia and Enamel Hypomineralisation

Enamel hypoplasia is a generic term defined as quantitative enamel defects that presents as foci of reduced enamel thickness. Classification of EH may be based on clinical appearance, pathogenesis, or etiology (Table 1).

**Table 1:** **Classification systems of enamel hypoplasia**.

**Enamel hypoplasia by clinical features**
Pitts
Lines/grooves
Areas
**Enamel hypoplasia by pathogenesis**
Hypoplastic enamel matrix production
Matrix hypomineralisation
**Enamel hypoplasia by etiologic factors**
**Hereditary factors**
Amelogenesis imperfecta
**Environmental factors**
Trauma
Infectious (i.e., CDV)
Pyrexia of any origin
Chemicals with toxic influence on ameloblasts (i.e., fluoride)

The Federation Dentaire International (FDI) classified EH in humans by its clinical appearance as either (1) *pits* (single/multiple, shallow/deep tiny areas of enamel loss), (2) *grooves* or lines of enamel loss (<2 mm wide), or (3) *areas* of partial or complete absence of enamel of a tooth crown (20).

Considering the process of amelogenesis, the pathogenesis of clinical EH may broadly be divided into two groups: (1) hypoplasia of the enamel due to incomplete or disrupted enamel matrix production and (2) hypoplasia as the consequence of inadequate matrix mineralization. Intact, hypomineralized defects initially present as demarcated opacities with clear borders, but as soon as the structurally weaker hypomineralised enamel chips away from the surrounding normal enamel, it may become impossible to distinguish it from hypoplasia due to inadequate matrix production (21). Nevertheless, in hypoplasia by reduced or inadequate enamel matrix formation, the borders to the normal enamel are generally regular and smooth, whereas in enamel substance loss due to reduced mineralization the enamel edges are initially sharp and irregular where the enamel has chipped off, sometimes later being smoothed by wear. By gently running a sharp dental explorer across the margins of the enamel defect, a distinction between the above visual assessments can usually be confirmed (21). Due to the complexities of differentiating EH on the ground of its pathogenesis, the terms EH and hypomineralisation will be combined here to describe clinical defects of enamel presenting with structural irregularities. These defects may occur as single tooth or focal pathology [focal enamel hypoplasia (FEH)] or affect the dentition diffusely [diffuse enamel hypoplasia (DEH)].

Etiologic factors that result in EH may be divided into two broad categories namely hereditary and environmental with the latter by far the most commonly encountered in clinical veterinary practice (Table 1). The clinical implications of enamel lesions as a result of any form of EH include the possibility of tooth sensitivity and increased susceptibility to wear, erosion, and even caries due to the structurally defective enamel and resultant more plaque retentive nature of the tooth surface. For the purpose of this manuscript EH will be discussed according to the extent of involvement of the dentition.

##### Focal Enamel Hypoplasia

**Figure 2:**
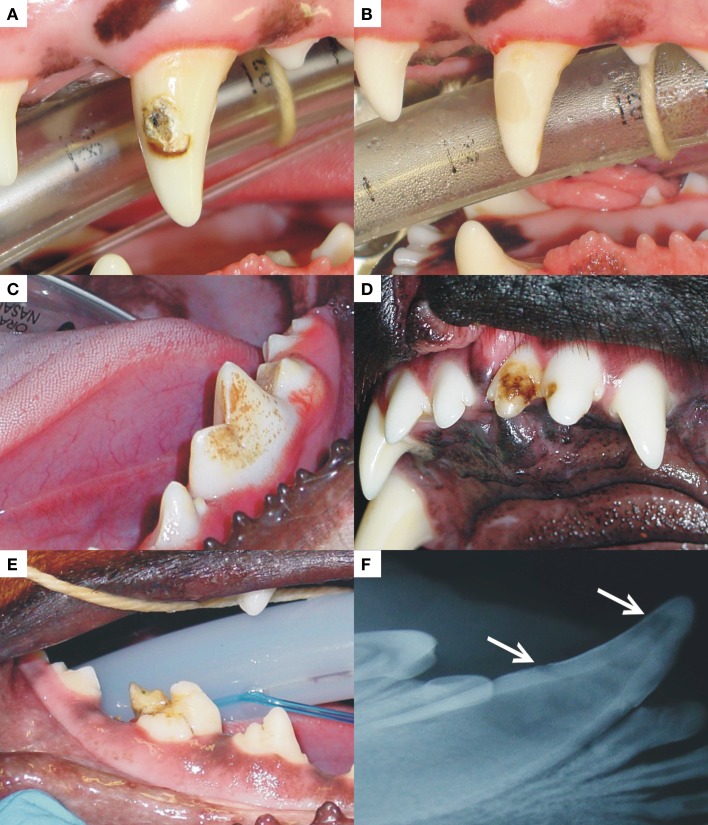
**A left maxillary canine (A) and left mandibular first molar (C) present with FEH, affecting the labial/buccal surfaces, respectively**. The photograph in **(D)** is an example of FEH affecting two adjoining maxillary incisor teeth and **(E)** a right mandibular first molar with enamel hypoplasia that is so extensive that the distal cusp is completely distorted. The radiograph in **(F)** represents the enamel changes seen in a case of FEH. Radiographic changes may be so subtle that they can go unnoticed. The photograph marked as **(B)** was taken after restoration with a dental compomer of the tooth shown in **(A)**.

Focal enamel hypoplasia, also known as Turner’s hypoplasia (22) after the human clinician who described this abnormality in 1912, is the most common type of dog tooth abnormality seen in the veterinary clinical practices of the authors. It refers to a clinical tooth defect varying from focal areas of opaque white, yellow, or brown discoloration of visibly abnormal enamel (Figures 2A,B) to grossly abnormal tooth morphology (Figure 2C). Often only one tooth in the mouth is affected and is referred to as a Turner’s tooth (22). Environmental rather than genetic factors are responsible for this focal form of EH, which most commonly follows localized infection or trauma involving a developing tooth germ. Due to the topographical relationship of a deciduous tooth with the developing permanent tooth, infection of or trauma to the deciduous tooth may result in EH of the permanent successor. Bite wounds during the first 8–10 weeks of life are suspected to be the most common reason for FEH in the authors’ practices. Turner’s tooth can be caused iatrogenically, commonly seen following poorly performed extraction of deciduous teeth. The pattern of EH seen in FEH is explained by the possibility of an insult that stopped some ABs producing enamel matrix whereas others were still able to complete their job (23). On the more extreme side of the scale, trauma to the early developing tooth germ may cause structural disturbance of the AB layer with complete change in the form of the developing tooth crown (Figures 2C–E). Radiographically, FEH may show foci or bands of less dense enamel on the affected tooth (Figure 2F). Due to the naturally thin nature of tooth enamel in dogs, however, these defects are not always clearly visible on radiographs.

Management of these defects varies from improved tooth brushing techniques to prevent plaque accumulation on the rough surface of the defect to restoration thereof (Figure 2B). In cases where the defect extends below the gingival margin, however, gingivitis is common around the affected teeth. If left untreated, this may increase the risk for periodontitis around these teeth. In cases where subgingival involvement prevents optimal restoration of the defect and where bone loss is already present, periodontal surgery prior to restoration or extraction is warranted.

##### Diffuse Enamel Hypoplasia

**Figure 3:**
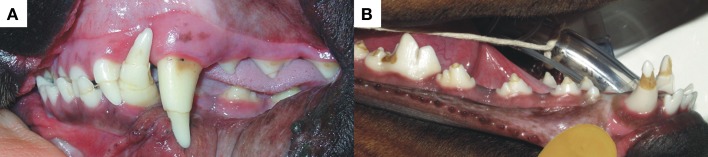
**The photographs represent two examples of DEH in different patients with circumferential hypoplastic lines present on almost all the teeth in (A)**. There is, however, still enamel present on all the crown surfaces, thus the unstained appearance. More severe enamel hypoplasia with dentin exposure and resultant staining is shown in **(B)**. The lesions must have been caused by systemic insults such as infectious disease with episodes of severe pyrexia associated.

Diffuse enamel hypoplasia presents with similar clinico-pathologic changes as described for FEH but with involvement of much of the dentition (Figure 3). Whereas FEH most commonly involve a localized insult to the jaw, DEH is usually the result of systemic diseases with pyrexia or direct infection of the actively enamel-producing ABs by microorganisms. The extent, duration, and time of pyrexia needed to produce various patterns of DEH have not been described in the veterinary literature. Canine distemper virus (CDV) infection, due to the epitheliotropic nature of the virus, is well known to produce DEH with direct infection and destruction of the ABs (24, 25) in addition to any effects of associated fever. Immunization has fortunately reduced the number of CDV-related DEH cases in many countries. The clinical pattern of enamel defects in DEH may present as pits, lines, or areas (20) that affect several teeth in all four jaw quadrants. An excellent publication that explains the micro-pathogenesis of each type is available (19). In the experience of the authors, DEH in dogs often presents as areas with circumferential involvement of the entire crown rather than only pits or lines (Figure 3). The extensive clinical involvement of many teeth in some dogs is hypothesized to be related to the relatively large amount of enamel formed within a relatively brief timespan as mentioned previously (15), resulting in larger defects in dogs when amelogenesis is interrupted or stopped for even just a few days.

Due to its extensive nature, management of DEH is more complex than in the case of FEH. Depending on the extent of hypoplasia, treatment will vary. Removal of soft and flaking enamel using a white carborundum stone followed by dental scaling, polishing, and fluoride treatment or application of a dental varnish to prevent sensitivity are both acceptable. When dentin becomes exposed, the teeth can become sensitive, particularly in recently erupted, immature teeth. In such cases, application of a dentin sealant or bonding agent dramatically improves comfort. Application of stannous fluoride by the owner should be carefully monitored, as toxicity resulting from overzealous application is a possibility. In all instances, optimal oral hygiene should be recommended to the owner to prevent carious decay of the hypoplastic areas.

#### Amelogenesis Imperfecta

**Figure 4:**
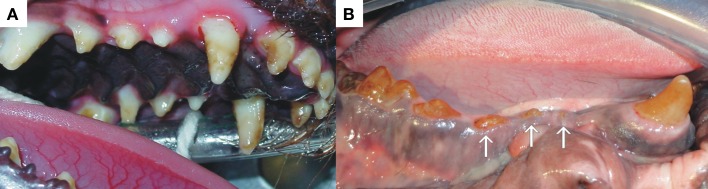
**Both patients (A,B) presented with extensive enamel hypoplasia affecting the entire dentition with obvious loss of dental structure (arrows)**. One can appreciate the involvement of all the surfaces of all the teeth with a variety of defects and color.

Amelogenesis imperfecta (AI) is a type of hereditary EH with a genetic, although non-syndromic, basis. The condition typically affects all teeth in the deciduous and/or permanent dentitions of humans, usually in a more uniform manner than the environmental EH described earlier. There is a wide variety of clinical presentations of AI with regard to the quantitative and qualitative appearance of enamel is concerned. These enamel features occur in the absence of any systemic, metabolic, or other diseases that could have caused such dental abnormalities. The pathogenesis of AI is complex, and classification systems are cumbersome. Most classification systems are based on phenotype, mode of inheritance, and the molecular basis of the genetic abnormality (26). Similar to environmental forms of EH, clinical classification systems are based on the process of amelogenesis and consider enamel matrix secretion, matrix mineralization, and enamel maturation. There are three clinically distinguishable types of AI: (1) a *hypoplastic type* with defective matrix secretion by the ABs, (2) a *hypocalcified type* where there is defective mineralization of the matrix, and (3) a *hypomature type* where enamel crystal growth during maturation is defective due to ineffective enamel protein removal (27). Genes relating to all three types have been recognized (28). Although AI is frequently included in textbooks and manuscripts on EH in dogs, sometimes in a confusing manner (29), only two studies with confirmed cases of AI in dogs are available in the English literature. One group described a familial-type EH in Standard Poodles in Sweden clinically and histologically resembling hypocalcified type AI (30) but without genetic confirmation. The study included a four-generation pedigree, and a research project aimed to determine the incidence of familial tooth discolorations in Swedish Poodles was undertaken. Over 2 years, 16.1% of 62 Swedish Poodles were confirmed to have similar dental changes (30). Another group reported a familial enamel hypoplasia uniformly affecting deciduous and permanent teeth in Italian Greyhounds. The clinical presentation closely resembled AI, and genetic analysis confirmed the presence of a 5-bp deletion in exon 10 of the *enamelin* (*ENAM*) gene (31). This genotype relates mostly to the hypoplastic type of AI (28). In humans, the clinical manifestations of AI usually depend on the type of AI present. In the genetically confirmed case of AI in the Greyhounds, the enamel was thin, rough with brownish mottling, and the teeth were small and pointed with larger than normal interdental gaps (31). No confirmed case of AI has been encountered by the authors in over two decades of referral practice, although at least four patients presented with rather homogenous EH highly suggestive of AI (Figure 4). The owners were advised of the presumptive diagnosis of AI, but as none permitted genetic or microscopic examination and on inquiry no related animal were identified as being affected, it was not possible to confirm the diagnosis of AI in any of these cases. Depending on the extent of hypoplasia caused by AI, treatment will vary. Most importantly, however, genetic counseling with recommendation to neuter, the genetically affected dog is recommended.

#### Developmental Tooth Discoloration

The color of teeth is influenced by several intrinsic structural as well as extrinsic factors (32). Understanding the mechanisms of tooth discoloration makes it easier to decide on treatment options, if needed at all.

Extrinsic stains on tooth enamel are the result of various types of pigments deposited on the enamel surface. They can usually be removed easily by scale and polish procedures. Although a description of extrinsic tooth discoloration is beyond the scope of this article, it is important to know that extrinsic stains may become incorporated in the tooth substance, an entity known as internalized discoloration. The latter is most commonly the result of structural enamel defects, either through developmental abnormalities of acquired defects following tooth wear, gingival recession, caries, or restorative materials (33). In these cases, a variety of chromogens can enter the porous defects or open dentinal tubules, resulting in discoloration of the tooth, which cannot be removed by normal scale and polish procedures. The affinity of chromogen for the dental structure varies with the type of chromogen and the strength of adhesion to the tooth structure, a process which is not clearly understood (34).

The intrinsic color of a tooth is influenced by the thickness and structural properties of the enamel, which influence the scattering and absorption of light within enamel. Due to the relatively translucent nature of the enamel, structural properties and color of the underlying dentin also play a major role in the overall tooth color (35, 36). A variety of local and systemic causes may result in intrinsic tooth discoloration. Systemic causes include drug-related tooth discoloration, notably tetracycline staining (see below), metabolic conditions such as fluorosis, and genetic conditions, which may result in tooth discoloration such as congenital erythropoietic porphyria with accumulation of porphyrins, hyperbilirubinemia, which causes deposition of byproducts of hemolysis, as well as structural defects such as AI and dentinogenesis imperfecta (33, 37). AI results in abnormal enamel structure with resultant large variety of clinical appearances and discoloration (see earlier). Dentinogenesis imperfecta on the other hand represents a range of rare genetic disorders that result in a variety of genetically abnormal dentin structure (27). The abnormal dentin may give inadequate support with resultant enamel fractures and exposure of porous dentin, prone to chromogen absorption. The dentin has an abnormal color, which results in opalescent teeth.

Local causes for intrinsic tooth discoloration are numerous and include pulp necrosis, pulpal hemorrhage (within the pulp cavity), and pulp tissue remnants after endodontic therapy, all resulting in deposition of hemoglobin-related pigments in the dentin. Endodontic materials within in the pulp chamber as well as aging with secondary and tertiary dentin deposition on the pulp walls also cause substantial tooth discoloration (33, 37). It is important for the clinician to distinguish between developmental and acquired intrinsic tooth discolorations in order to apply the correct treatment when so indicated, mostly in the case of acquired discoloration.

##### Tetracycline Staining

**Figure 5:**
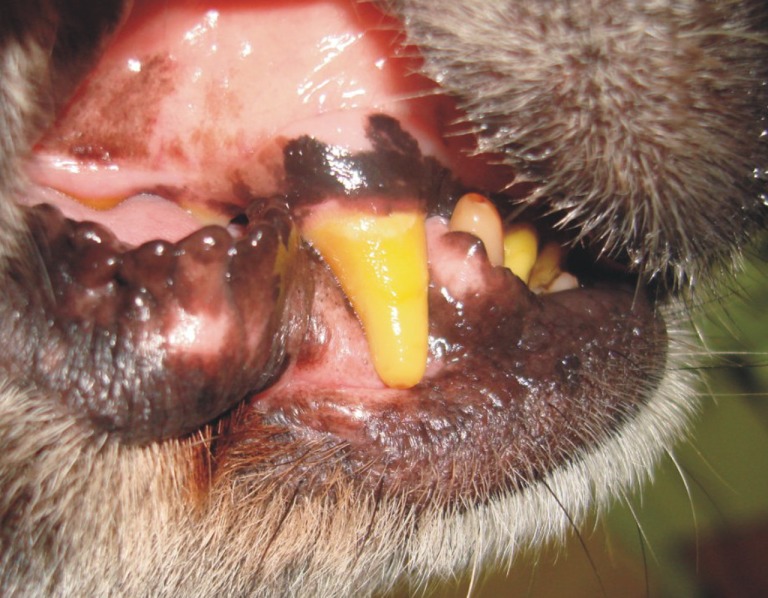
**The photograph is an example of the bright-yellow discoloration of the teeth in a patient that was on confirmed tetracycline treatment as a puppy**. Apart from the discoloration, there were no structural defects of the enamel on clinical examination.

Tetracycline has been on the market for more than six decades and is commonly used for the treatment of a range of bacterial, chlamydial, mycoplasmal, and rickettsial infections. One of the most adverse drug reactions of tetracycline usage is permanent yellow to brown tooth discoloration (38), affecting the parts of the teeth that were in the process of matrix mineralization and maturation at the time of usage (39). Due to this undesirable side effect, tetracycline should not be administered to pregnant bitches or puppies <6 months of age (40, 41). Dosage, time of exposure to the drug, and stage of tooth mineralization at the time of usage will determine or influence the degree of tooth discoloration. Tooth staining also occurs with use of a number of derivatives of tetracycline including doxycycline and minocycline, among others, though the staining is typically less dramatic (42, 43). Tetracycline binds irreversibly to and forms complexes with calcium orthophosphate in the affected teeth, which darkens with exposure to light due to oxidation (39, 43). The pathogenesis of minocycline staining is still uncertain, and several hypotheses are available in the literature (43). It is, however, not commonly used in veterinary medicine, unlike the situation in human medicine where it is often used to treat dermatological conditions. The authors have only seen two confirmed cases of tetracycline staining in dogs over the last two decades (Figure 5). Tetracycline staining, although esthetically displeasing, produces no structural weakness of the dental hard tissue, and no treatment is necessary in affected dogs.

### Developmental Abnormalities of Macroscopic Tooth Anatomy

#### Double Teeth

**Figure 6:**
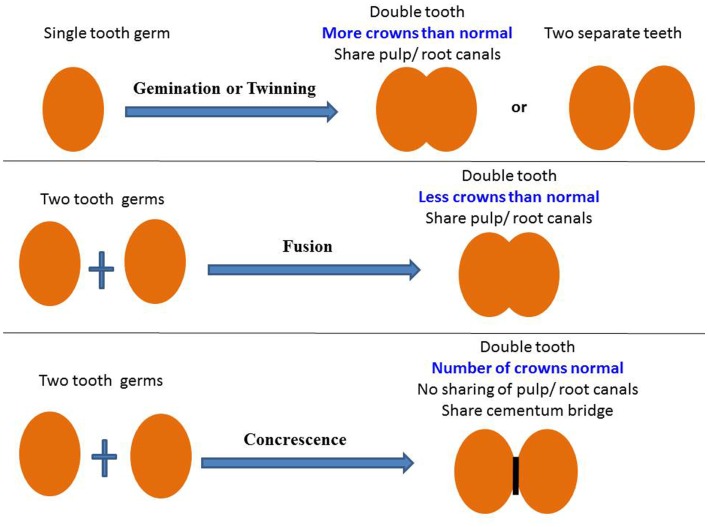
**The diagram presents a summary for the definitions of gemination, twinning, fusion, and concrescence**.

“Double teeth” is a common term used to describe any situation where two teeth are joined together by dental hard tissue in the form of enamel, dentin, and/or cementum regardless of whether or not they share part of or even the entire pulp cavity. There are several developmental conditions that fall within this category, namely gemination, twinning, fusion, and concrescence, each implying a certain pathogenesis. Confusion exists on how to reliably distinguish between these dental anomalies in clinical practice, irrespective of how experienced the clinician is. Some modern researchers prefer a neutral term such as “double teeth” to refer to all of these abnormalities (4). For the purpose of this manuscript and due to the fact that the separate terminologies are still commonly used in the dental literature, the authors will try to present clinical guidelines as to the different entities within this group of developmental abnormalities (Figure 6).

##### Gemination

**Figure 7:**
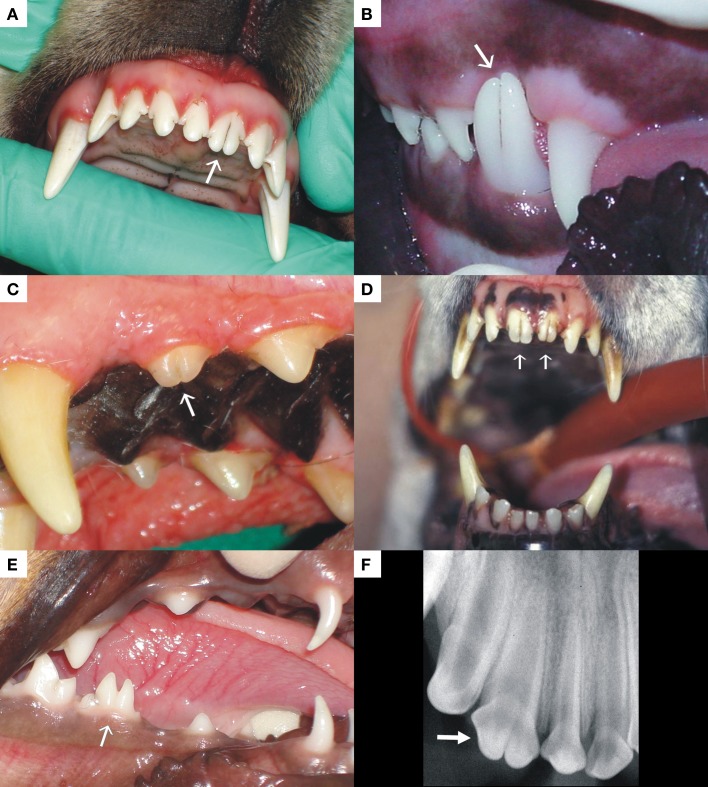
**The photographs exhibit examples of gemination encountered in an incisor (A), canine (B), and premolar tooth (C) of dogs**. **(D)** represents a case of bilateral permanent maxillary first incisor tooth gemination, and **(E)** was a case of gemination of a deciduous premolar tooth in a Dachshund. **(F)** is a radiograph of a geminated second maxillary incisor tooth demonstrating a single root with two crowns.

The term gemination, also known as *schizodontism*, refers to the attempted endeavor of a single tooth germ to divide into two separate teeth. Only partial division occurs, resulting in a single, larger than normal, tooth with a bifid crown (44) (Figure 6). If gemination was successful, there would be an extra (supernumerary) tooth in the dental arch (this complete separation being termed “twinning”). With gemination, the number of teeth remains normal if the large abnormal tooth is regarded as a single tooth. One of the oldest manuscripts to describe tooth gemination in dogs proposed that gemination could be the result of connation or fusion between a normal tooth germ and that of a supernumerary or extra tooth (45). The initial thought of apparent gemination being fusion between the germs of a normal and a supernumerary tooth is a reality in some cases and well described in the human dental literature (46). One way to clinically distinguish between the two possibilities is that a supernumerary tooth which is often cone-shaped or anatomically abnormal will, when fused with a normal tooth, result in a large tooth with differences between the two halves of the joined crown. In true gemination, the two halves of the joined crown are anatomically similar and often mirror images of each other with a groove that extends between the two teeth through the incisal edge of the crown. Gemination in dogs is mostly seen in an incisor, canine, or premolar tooth (47), but molars can also be affected with multiple affected teeth being reported in a human patient, sometimes with bilateral symmetry (48). Cases such as these have never been described in the veterinary literature. Gemination has been described to occur with increased frequency in Boxers compared with other breeds of dogs (1, 49). In the authors’ clinical experience, the incidence of gemination is low, but cases involving canines, premolars, and most commonly incisor teeth are regularly encountered (Figures 7A–F). Over the last 20 years, three cases of bilateral gemination were seen, one affecting the canine teeth in a Border Collie, one the maxillary first incisors of a medium-sized cross-breed dog (Figure 7D), and another affecting the mandibular third incisor teeth in an Irish setter. The human literature describes gemination to occur most frequently in the deciduous dentition (50, 51), but in veterinary referral practice gemination is most frequently encountered in permanent teeth. Only one case of deciduous gemination was encountered between the two referral practices, where a Dachshund’s right mandibular deciduous premolar tooth was affected (Figure 7E). The etiology for gemination is thought to be multifactorial, with genetic and environmental causes described by different authors (1, 49, 52, 53).

The crown of a geminated tooth does not usually require any treatment other than awareness of the lesion and optimal oral hygiene. In the case where the groove between the two crowns extends subgingivally, bacterial plaque accumulation is common and can result in chronic gingivitis and potentially periodontitis. Sealants and resin restorations placed carefully in these grooves may reduce the risk of inflammation, but in some cases, gingivoplasty is a more practical method to prevent periodontitis-related bone loss. Gemination may also result in poor tooth alignment leading to crowding of teeth, with all its consequences, soft or hard tissue damage, when teeth occlude against soft tissue or opposing teeth, and delayed tooth eruption.

##### Fusion

**Figure 8:**
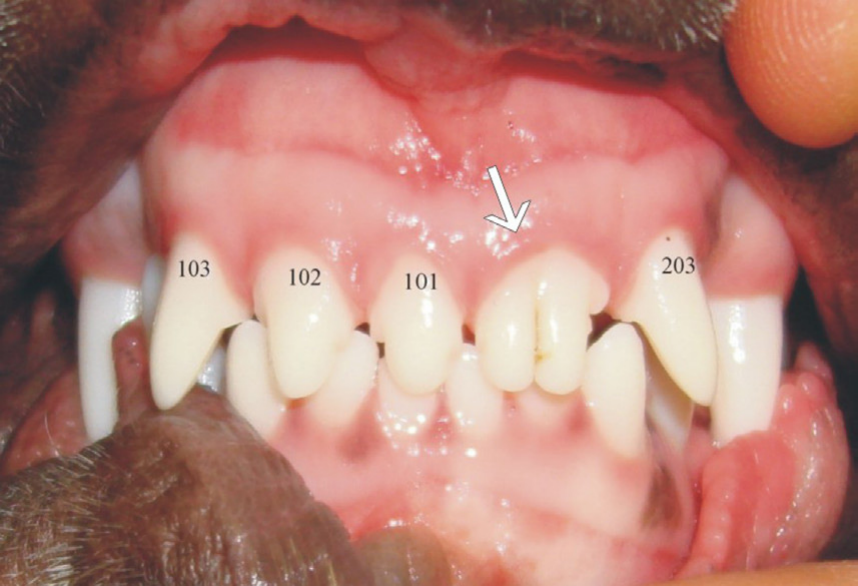
**The patient presents with a good example of fusion between teeth 201 (left maxillary first incisor) and 202 (left maxillary second incisor) resulting in a double tooth in their place**.

The term fusion is used to describe a double tooth similar in clinical appearance of a geminated tooth, but when the number of teeth is counted, one tooth is missing from the dental arch. However, fusion could also occur between a normal and a supernumerary tooth resulting in a normal number of teeth. Fusion results when two separate tooth buds unite at the crowns and/or roots before hard tissue formation is completed (Figure 6). A large, sometimes double tooth is usually the result. It may involve only the crown or extend along the entire length of the tooth, from crown to apex. Fused teeth will usually present radiographically with separate root canals. Fusion in dogs have been described infrequently (1, 49, 54), and only two cases have been seen by the authors in the last 20 years (Figure 8). The etiology is unknown, but the possibility of a physical force bringing two developing teeth close with each other, so that the epithelial layers come into contact and fuse with one another, has been postulated (55). A genetic predisposition has also been reported; as a possible contributing factor, as fusion of teeth is also seen with higher frequency in human patients with achondrodysplasia, chondroectodermal dysplasia, focal dermal hypoplasia, and osteopetrosis (56). The possibility of a familial tendency in dogs has also been postulated (54), although its association with any specific genetic condition has not been described.

The management principles of fused teeth in dogs are similar to those described for gemination.

#### Concrescence

**Figure 9:**
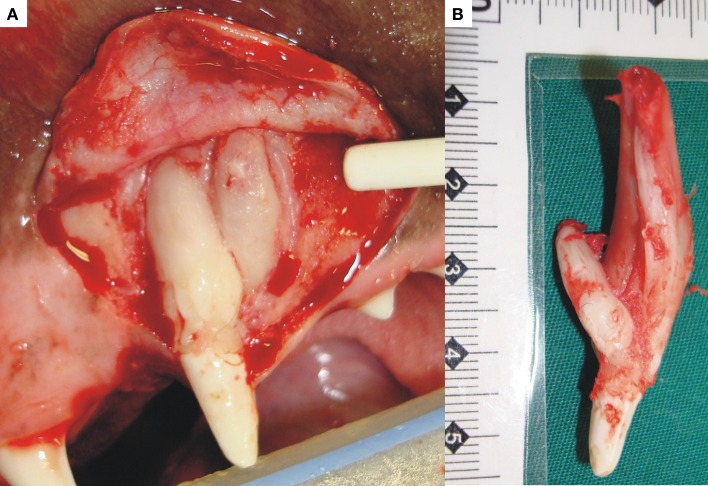
**The photograph (A) shows an example of concrescence between a canine and supernumerary tooth in the left maxilla**. The pathogenesis in this case was uncertain. Both were surgically removed. On examination of the excised teeth **(B)**, they shared only a cementum bridge but not dentin or any other dental hard tissue.

Concrescence differs from gemination and fusion due to the fact that it describes union of two or more fully formed teeth by only their cementum with no involvement of dentin or enamel (Figure 6) (57). The area of fusion may only involve a small area, while in other cases it can occur as a solid mass of cementum extending along the entire root surface (58). Concrescence may be developmental (also known as “true” concrescence) or be acquired as a result of inflammation-induced hypercementosis (57). In the case of true concrescence, it is hypothesized to be the result of trauma or even crowding of teeth with abnormal resorption of interproximal bone and resultant cementum deposition between the adjacent roots (59). In the case of acquired concrescence, inflammation might result in the interproximal bone resorption with the same result (58). This type of cementum fusion affecting teeth has been infrequently described in dogs particularly between canine and first premolar teeth (1, 47, 60). The appearance may be difficult to interpret on two-dimensional radiographs, appearing as overlapping or superimposition of two teeth or even be misinterpreted as close proximity of the roots of adjacent teeth. Three-dimensional analysis techniques such as cone-beam computed tomography (CBCT) and micro-CT provide improved diagnostic imaging results for concrescence (61), and it has been recognized as an incidental finding by one of the authors (David Crossley) on standard CT imaging of a dog’s jaw. Teeth fused at the cementum level may pose problems during extraction, and prior knowledge regarding this developmental condition is helpful in surgical planning. Only one case of concrescence between a canine and a supernumerary tooth was seen in our clinical practice (Figure 9). The diagnosis in that case was histologically confirmed on decalcified hematoxylin and eosin-stained sections, and the two teeth were surgically removed with no complications.

#### Dens Invaginatus

**Figure 10:**
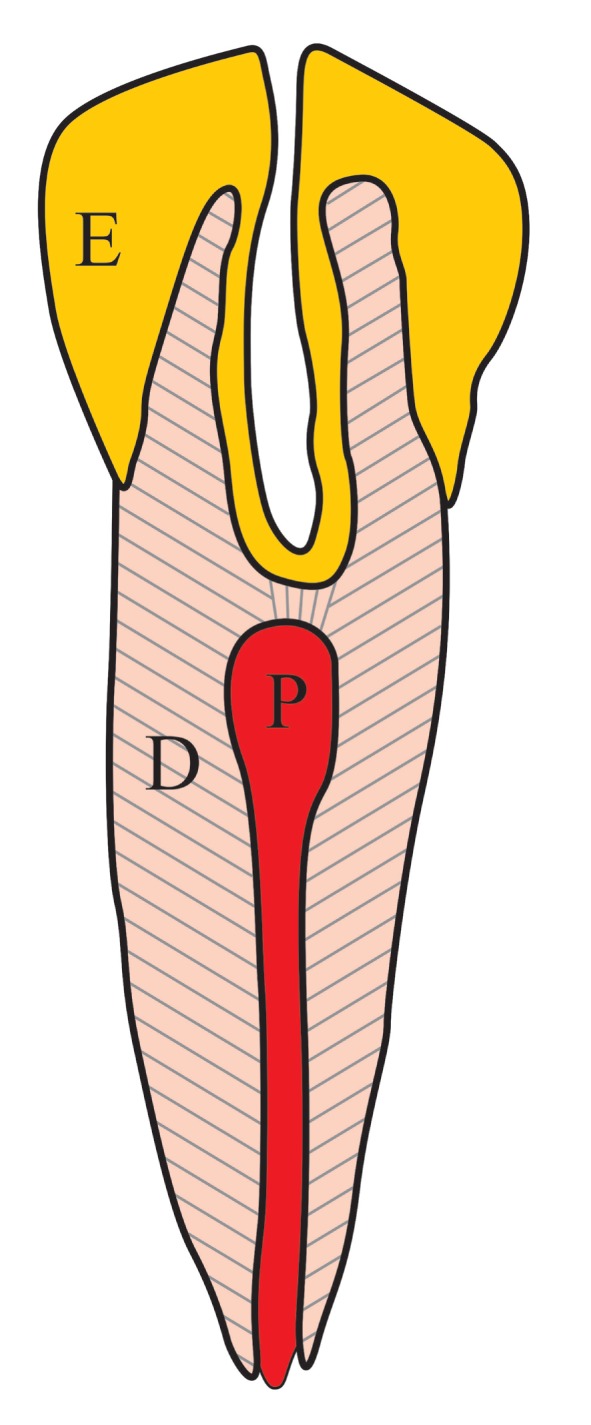
**The diagram aims to explain the pathogenesis of a *dens invaginatus* tooth**. During development, there is invagination of the inner enamel epithelium into the tooth pulp (P), resulting in a deep infolding of enamel (E) into dentin (D) and pulp (P). Radiographically, this may give the impression of a tooth within a tooth.

*Dens invaginatus* (DI) is also known as *dens in dente*, tooth within a tooth, invaginated odontome, tooth inclusion, dilated odontoma, and dents telescopes, with other descriptions and names also being used from time to time. It is an interesting developmental abnormality that results when the developing tooth crown surface invaginates into the developing tooth pulp before mineralization has occurred (Figure 10). The result is a crown that varies from being normal to teeth with increased diameter, strange shapes, or extra cusps (62, 63). The etiology and pathogenesis is mostly speculative and includes theories of pressure on or trauma to the developing tooth, focal failure, or excessive proliferation of the internal enamel epithelium or a form of fusion between two tooth germs (64, 65). Radiographically, DI sometimes has the appearance of a small tooth-like structure within the pulp cavity of another tooth. Depending on the extent of invagination into the tooth pulp, DI is classified as type I (invagination in the crown only, which does not extend beyond the cementoenamel junction), type II (invagination extends beyond the cementoenamel junction into the root canal and ends as a blind sac), and type III (invagination extends through the root or lateral surface to form an additional opening or foramen but with no direct communication with the existing pulp canal) (64). This abnormality is uncommon, and only a few case reports on DI in dogs are found in the literature (62, 66, 67).

Due to the fact that DI involves the dentin–pulp complex of the affected tooth, secondary infection of the pulp may follow, which ultimately leads to endodontic-periodontic pathology with bone loss (49). In addition, the abnormal tooth morphology of a type III DI may disturb the normal gingival contour at the furcation area of a multi-rooted tooth or along the root surface, predisposing the tooth to develop periodontitis. Treatment is therefore dependant on the extent of both the morphological pathology present and any associated secondary pathology. In some cases, restoration of deep surface indentations is possible, together with endodontic treatment when indicated, but in severe cases, extraction of the affected tooth is the only practical option (68).

#### Dilaceration

**Figure 11:**
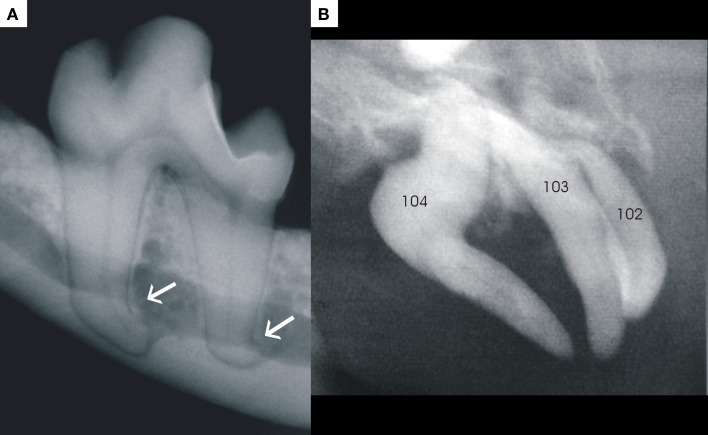
**Photograph (A) demonstrates an example of dilacerations of both roots of a left mandibular first molar, and photograph (B) is a good example of dilaceration of the crown of a right maxillary canine (tooth 104)**.

Dilaceration is a developmental abnormality where there is a sharp bent or curve anywhere along the root or crown of a formed tooth. An exact definition as to the degree of curvature needed before making a diagnosis of dilaceration varies from more than 20° to 90° (69). Dilaceration should be differentiated from root angulation, which is a more gradual change in the developmental direction of the developing tooth (70). Dilacerations of the tooth roots are more common than dilacerations of the crown (71). Acute mechanical trauma with intrusion of a deciduous tooth and the resultant physical damage to the developing permanent tooth is the most common theory regarding the etiology of dilacerations (72). According to this theory, the already mineralized and harder part of the developing tooth is displaced relative to the softer non-mineralized part, which then continues developing. There is, however, a large proportion of dilacerated teeth with no clear history of trauma, and these cases are considered to be idiopathic (73). Several other hypotheses regarding dilaceration have been postulated in the human literature (69, 74), but this phenomenon is less commonly reported in the veterinary literature (1). Dilaceration of roots also was commonly found in dogs affected by X-linked hypohidrotic ectodermal dysplasia (75). The authors have encountered more cases of root dilaceration (Figure 11A) than coronal dilacerations (Figure 11B). None of the root dilacerations posed any clinical difficulties, while three of the four teeth with coronal dilacerations required extraction due either to malocclusion or gingival compromise. Root dilaceration mostly does not pose clinical problems for the patient, but may cause difficulties during root canal treatments and extraction of such teeth.

#### Enamel Pearls (Enamelomas)

These represent small nodules of enamel, usually <4 mm, located on the root surface of the tooth close to or at the cementoenamel junction. They most commonly occur at the bifurcation or trifurcation of molars and are rare on incisor teeth. The incidence of enamel pearls in humans is approximately 5% (76, 77), but its occurrence in dogs is uncertain and should be investigated (78). Microscopically, the enamel pearl consists of a core of dentin covered by enamel and may contain a pulp chamber continuous with the larger pup chamber of the tooth. The etiopathogenesis of enamel pearls remains uncertain, as differentiated ABs below the cementoenamel junction are needed for its development (79). The exophytic projections, radiographically visible on the root surfaces, may result in plaque accumulation, thus favoring the establishment and progression of periodontal disease (80). Even so, attempts at removal of enamel pearls usually will do more harm than good.

## Conclusion

Developmental tooth abnormalities in dogs are rarely reported in the literature. Most of these abnormalities also were rarely encountered between two veterinary dental referral practices, which see more than 900 dental referral cases per annum between the two practices. FEH, while still uncommon, remains the most frequently encountered form of developmental tooth abnormality in the authors’ experience. Focal trauma to a developing tooth, most likely as a result of bite wounds from another dog seems to be the most plausible etiologic factor in this regard. Future research should aim to provide a detailed individual tooth mineralization schedule for dogs which could give a much better indication as to the pathogenesis of developmental tooth abnormalities when they do occur. It is also proposed that publications on possible cases of AI should be supported by genetic studies in order to determine the true incidence of this very rare condition in dogs. Other developmental defects preferentially involving the pulp, dentin, and cementum may also occur and can be associated with abnormalities of the enamel and tooth crown. Developmental tooth abnormalities, although rare may still pose clinical challenges with regard to diagnosis and treatment. Their correct recognition, diagnosis, as well as education of clinicians on proper instrument selection and placement during primary tooth extraction remain the most important aspects in the management of these lesions.

## Author Contributions

All authors listed, have made substantial, direct, and intellectual contribution to the work and approved it for publication.

## Conflict of Interest Statement

The authors declare that the research was conducted in the absence of any commercial or financial relationships that could be construed as a potential conflict of interest.
